# Chromosome-scale genome assembly of *Lepus oiostolus* (*Lepus*, Leporidae)

**DOI:** 10.1038/s41597-024-03024-6

**Published:** 2024-02-10

**Authors:** Shuo Feng, Yaying Zhang, Zhaotong He, Erning Xi, Dafu Ru, Jian Liang, Yongzhi Yang

**Affiliations:** 1 State Key Laboratory of Plateau Ecology and Agriculture, Qinghai University, Xining, 810016 China; 2 State Key Laboratory of Grassland Agro-Ecosystems, and College of Ecology, Lanzhou University, Lanzhou, 730000 China

**Keywords:** Genome assembly algorithms, Evolutionary ecology, Zoology

## Abstract

*Lepus oiostolus* (*L*. *oiostolus*) is a species endemic to the Qinghai-Tibet Plateau. However, the absence of a reference genome limits genetic studies. Here, we reported a high-quality *L*. *oiostolus* genome assembly, with scaffolds anchored to 24 chromosomes and a total assembled length of 2.80 Gb (contig N50 = 64.25 Mb). Genomic annotation uncovered 22,295 protein-coding genes and identified 49.84% of the sequences as transposable elements. Long interspersed nuclear elements (LINEs) constitute a high proportion of the genome. Our study is at the first time to report the chromosome-scale genome for the species of the *L*. *oiostolus*. It provides a valuable genomic resource for future research on the evolution of the Leporidae.

## Background & Summary

The emergence and rapid development of new sequencing technologies, such as single-molecule real-time sequencing (SMRT) by Pacific Biosciences (PacBio)^1^, nanopore sequencing by Oxford Nanopore Technologies^2^ 10x genomics^3^, optical and chromosome-contact maps from BioNano Genomics^4^, and Hi-C sequencing^5^ have facilitated the construction of high-quality genome assemblies^6^. Integrative application of these techniques elevate human genome assembly to a Telomere-to-Telomere (T2T) gapless level. Subsequent mining of the human T2T genome unveils genetic variations of mitoplasts, centromeres and other previously unassembled regions, which significantly enriched the understanding of human genome diversity, evolution and disease occurrence^7^. Other studies on important livestock also showed the important values of high quality genome assemblies. Qiu *et al*. presented the draft genome sequence of a female domestic yak generated using Illumina-based technology and related to sensory perception and energy metabolism, as well as an enrichment of protein domains involved in sensing the extracellular environment and hypoxic stress^8^. Zhang *et al*. used long-read resequencing data for 6 wild and 23 domestic yaks and identified genes that are predominantly related to the nervous system, behaviour, immunity, and reproduction and may have been targeted by artificial selection during yak domestication^9^. Liu *et al*. constructed a graph-genome for 47 genomes of 7 cross-fertile bovine species and validated a significant association of the selected stratified SVs with gene expression^10^. These aforementioned research greatly deepened the understanding of the genetic basis of high-altitude adaptation of bovine species. Decoding of high-quality genome assemblies is increasingly becoming a powerful approach serving clarification of the genetic mechanisms of adaptive evolution.

*L*. *oiostolus* is a species endemic to the Qinghai-Tibet Plateau (QTP) and is also commonly known as the gray-tailed hare. It belongs to the genus *Lepus*^11^. It is distributed at an elevation of 2,100–4,000 m in the alpine zone of the Qinghai-Tibet Plateau in China, making it the highest-altitude rabbit species in the world. In contrast, the typical habitat of rabbits rarely exceeds 600 m at altitude^12^. For instance, *Oryctolagus cuniculus*, a closely related species of *L*. *oiostolus*, has been used as a model organism for biomedical research. Previous physiological studies demonstrated that *L*. *oiostolus* has developed unique tolerance to plateau environment, making this species an excellent model for investigation of genetic mechanisms contributing to high-altitude adaptation^13^. Although deciphering of genome assemblies of plateau animals have significantly broaden our knowledge horizon concerning plateau adaptation and evolution, the genome information of *L. oiostolus* is still lacking^8,14,15^.

In this study, we assembled a chromosome-scale genome of *L*. *oiostolus* by combining Illumina and PacBio data with Hi-C technology. The final genome size was 2.85 Gb with N50 sizes of 64.25 Mb and a complete Benchmarking Universal Single-Copy Orthologs (BUSCO) score of 96.2%. A total of 2.80 Gb (98.1%) genome sequences were further clustered and ordered into 24 chromosomes. Genome annotation predicted 22,295 protein-coding genes. The availability of a complete and detailed genome assembly is essential to basic biological research. This paper provides a valuable genomic resource for research into the molecular mechanisms and evolution of *L*. *oiostolus*.

## Methods

### Sample collection and sequencing

*L*. *oiostolus* samples for genome sequencing were collected from Haiyan County, Haibei Prefecture, Qinghai Province, China (100°98’E, 36°90’N). Genomic DNA was extracted from muscle tissue and blood using the TIANamp Genomic DNA kit. Agarose gel electrophoresis (0.7% agarose gel) was used to assess overall DNA quality. DNA purity was assessed using a NanoDrop One Spectrophotometer (Thermo Fisher Scientific), and DNA concentration was determined using a Qubit 3.0 Fluorometer (Life Technologies, Carlsbad, CA, USA).

We implemented a hybrid strategy combining Illumina short-read sequencing, PacBio long-read sequencing, and Hi-C sequencing technologies to obtain better sequencing data. For Illumina short reads, a genomic library with insert sizes of 150 bp was constructed using the NextEra DNA Flex X Library Prep Kit (Illumina, San Diego, CA, USA). Qubit 2.0, Agilent 2100, and qPCR were used to ensure the quality of the library, and then the library was subsequently sequenced on the Illumina NovaSeq. 6000 platform (Illumina, San Diego, CA, USA). For PacBio long-read sequencing, PCR-free SMRT bell libraries were constructed and sequenced on the PacBio Sequel II sequencing platform. To generate a chromosomal-level assembly of the *L*. *oiostolus* genome, a Hi-C library was generated by the DpnII restriction enzyme following *in situ* ligation protocol^14^. The formaldehyde cross-linked DNAs were digested with DpnII and treated with biotin, which were ligated to the ends of the fragmented DNA sequences. Cyclization of DNA after end-repair was used to identify the location of intersecting DNA. After reversing the crosslinking, ligated DNA fragments were fragmented into 300–700 bp sizes, followed by a biotin-streptavidin purification for library construction. Finally, the Hi-C libraries were quantified and sequenced with PE150 sequencing read lengths on the Illumina platform. Finally, 79.03 Gb of PacBio reads (Table S1) and 177.15 Gb of filtered Illumina short-read sequencing data (Table S2) were obtained from the *L. oiostolus* genome.

### Genome survey and assembly

**Fig. 1:**
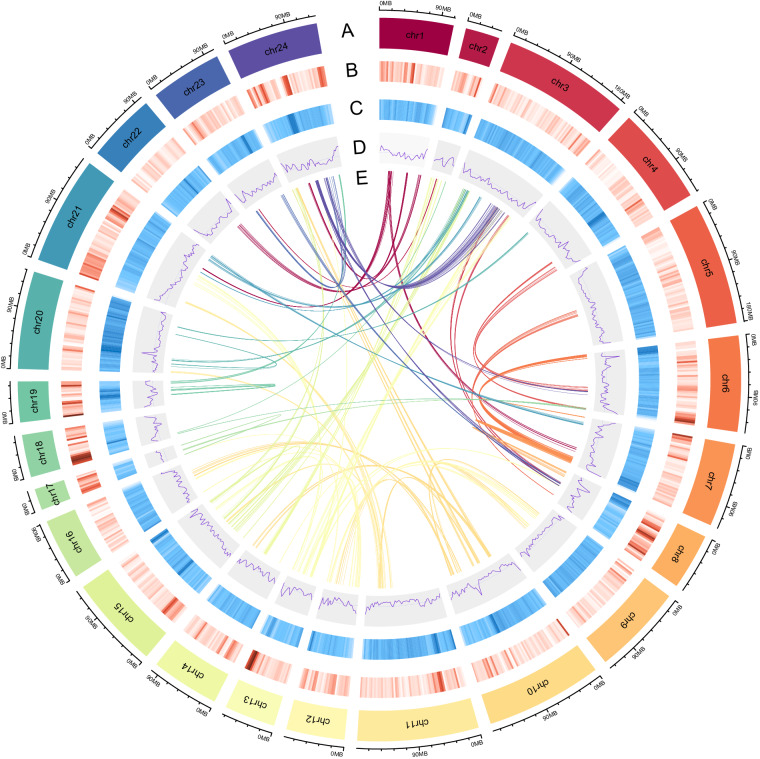
Distribution of various elements on the chromosomes of *L*. *oiostolus*. From the outer circle to the inner circle: (A) Chromosomes karyotype. (B) Gene density. (C) Distribution of GC content in the genome. (D) Repetitive elements density. (E) Schematic presentation of major inter-chromosomal relationships in the *L*. *oiostolus* genome.

Using the kmer-freq subroutine in the software GCE v1.0.0, the genome size, heterozygosity, and duplication ratio were estimated based on the k-mer distribution of 19-mers extracted from Illumina short reads. A total of 223.91 Gb raw PacBio subreads were filtered and corrected with the CCS pipeline v6.0.0 (parameters: -min-passes 3 –min-snr 2.5 –top-passes 60)^15^. The resulting CCS reads were subjected to hifiasm v0.14.2^16^ for de novo assembly. We corrected the primary contigs with the pilon v1.23^17^ using 89.12 Gb (70.35×) of Illumina paired-end reads. Using the samtools v1.9^18^ and bwa v0.7.17^14^, after which the processed reads from the Illumina sequencing platform were mapped to the reference genome. The mapping rate and coverage were counted to confirm the consistency and integrity of the assembled genome. The genome size is 2.83 Gb with 0.72% heterozygosity and 36.55% duplicate repeatability, as estimated by K-mer-based methods (Table S3; Figure S1). The resulting assembly was 2.86 Gb with a contig N50 of 64.25 Mb and the longest contig being 147.07 Mb in length (Table 1; Fig. 1). 99.99% of Illumina short reads were successfully aligned to our assembly (Table S4).

**Table 1:** Features of the assembled genome.

Genome assembly	
Total length (bp)	2,855,382,981
Contig N50 (bp)	64,246,700
Contig N90 (bp)	11,093,139
Contig number	344
Max length (bp)	147,072,656
Pseudo-chromosome length (bp)	2,800,548,853
Pseudo-chromosome nwumber	24
Anchored rate (%)	98.08
BUSCO evaluation	C: 96.2% [S: 93.3%, D: 2.9%], F: 0.9%, M: 2.9%, n: 1375

Note: Anchored rate refers to the percentage of contig sequences scaffolded into a pseudo-chromosome. C, Complete BUSCOs; D, Complete and duplicated BUSCOs; F, Fragmented BUSCOs; M, Missing BUSCOs; S, Complete and single-copy BUSCOs.

Hi-C analysis was used to generate the contig assembly at the chromosome level. The raw data were filtered using a perl script as implemented in the software LACHESIS v1.57^15^. To obtain uniquely mapped read pairs, the cleaned data were aligned to the initial genome assembly using BWA-MEM. Approximately 1.18 Gb of clean data were generated (Table S5). The hicup v0.8.0^16^ was used to evaluate the validity of the Hi-C data based on uniquely mapped read pairs. We only used valid read pairs for the draft genome re-correction and chromosome-level genome assembly. We further applied ALLHIC pipeline v0.9.12^17^ to link the contigs into 24 pseudo chromosomes. Finally, our team manually curated the Hi-C scaffolding based on the chromatin contact matrix in the Juicebox v1.11.08^18^. The scaffold assembly was obtained using the ALLHIC pipeline v0.9.12^17^ with 748.17 million uniquely mapped reads from cleaned Hi-C data (Table S6). A total of 2.80 Gb of sequences were anchored onto 24 pseudo chromosomes, accounting for 98.08% of the initial assembly (Figure S2, Table 2). In addition, Hi-C data were mapped against the Hi-C scaffold assembly, showing 74.31% valid sequences (Table S6). Genome-wide analysis of chromatin interactions showed a well-organized pattern of Hi-C signals along the diagonals, indicating a high-quality chromosomal genome assembly for *L*. *oiostolus* (Figure S2).

**Table 2:** Statistics of *L*. *oiostolus* genome sequence length (chromosome level).

Chromosome	Length (bp)	Percentage
1	109,229,759	3.83
2	50,538,581	1.77
3	186,833,085	6.54
4	144,762,858	5.10
5	180,929,746	6.34
6	141,402,830	4.95
7	123,451,712	4.32
8	83,315,911	2.92
9	133,999,074	4.69
10	172,682,257	6.05
11	179,416,224	6.28
12	86,613,829	3.03
13	75,545,960	2.65
14	100,844,688	3.53
15	137,608,147	4.82
16	91,128,452	3.19
17	31,644,398	1.11
18	62,955,406	2.20
19	60,076,877	2.10
20	144,192,792	5.05
21	160,215,235	5.61
22	102,004,348	3.57
23	105,886,210	3.71
24	135,294,474	4.74
Total	2,855,406,981	98.08
Unplaced	54,834,128	1.92

### Genome structure prediction and annotation

Both homology-based and de novo prediction methods were used to identify repetitive DNA elements. For the de novo prediction, RepeatModeler v1.0.11^19^ was used to construct an ab initio database of predicted repetitive elements, and RepeatMasker v4.0.9^20^ was used to annotate the repetitive elements in the database. RepeatMasker and RepeatProteinMask were then used to search the genome sequence for known repetitive elements, with the genome sequences used as queries against the RepBase database v27.06 (http://www.girinst.org/repbase). Tandem repeats were also identified with the TRF method. Repetitive elements play an essential role in genome evolution. In the repeat annotation, a total of 1,423, 017,427 bp of transposon elements (TEs) comprise 49.84% of the *L*. *oiostolus* genome. Among all the classifications of TEs, LINE constituted the largest portion (Table 3, Figure S3).

**Table 3:** Transposable element (TEs) annotations in *L*. *oiostolus*.

Type	TE proteins		De novo + Repbase		Combined TEs	
	Length (bp)	% in genome	Length (bp)	% in genome	Length (bp)	% in genome
DNA	2,980,913	0.10	85,281,607	2.99	86,850,625	3.04
LINE	183,928,802	6.44	615,678,820	21.56	622,457,755	21.80
SINE	0	0.00	495,976,943	17.37	495,976,943	17.37
LTR	14,650,912	0.51	137,218,044	4.81	139,623,355	4.89
Satellite	0	0.00	82,417,324	2.89	82,417,324	2.89
Simple repeat	0	0.00	0	0.00	0	0.00
Other	546	≈0	910	≈0	1,456	≈0
Unknown	11,958	0.00	76,813,774	2.69	76,825,732	2.69
Total	201,547,236	7.06	1,387,639,381	48.60	1,423,017,427	49.84

Note: TE proteins were identified using RepeatProteinMask against Repbase proteins; De novo TEs were identified using RepeatMasker in combination with the de novo library produced by RepeatModeler; Repbase TEs were identified using RepeatMasker with the Repbase library; Combined TEs: the combined results of these three discovery steps.

Genome structure analysis was conducted using homology-based prediction, de novo prediction, and RNA-seq-based prediction. For homology-based prediction, the amino acid sequences from *Aotus nancymaae*, *Callithrix jacchus*, *Carlito syrichta*, *Ochotona princeps*, and *Ochotona curzoniae* were aligned to the *L*. *oiostolus* assembly by using Exonerate v2.4.0^21^. For de novo gene prediction, Augustus v3.3.2^22,23^, Genscan v1.0^24^, and GlimmerHMM v3.0.4^25^ were used to predict coding regions in the genome with internal gene models. We used Stringtie v2.1.1^26^ to align assembled transcripts to the *L*. *oiostolus* genomic sequence and then TransDecoder v5.1.0^24^ from the Trinity package to identify likely open reading frames within the transcripts. Finally, the gene sets obtained from the predictions of various methods were integrated using Maker v2.31.10^25^. After the characterization of repeat sequences, we used Maker v2.31.10^25^ to predict a consistent set of genes with 24,410 total genes annotated. Overall, 251 complete BUSCOs (98.4%), including 239 single-copy (93.7%) and 12 duplicate BUSCOs (4.7%), and one fragmented BUSCO (0.4%) were identified in the gene annotations (Table 4). Three genes (1.2%) were recognized as missing BUSCOs in our genome (Table 4).

**Table 4:** Summary statistics of predicted protein-coding genes.

Gene set		Number	Average gene length (bp)	Average CDS length (bp)	Average exons per gene	Average exon length (bp)	Average intron length (bp)
De novo	Genscan	50,448	33,164	1,378	7.94	173.56	4,577
	Augustus	35,591	24,725	1,124	6.26	179.63	4,487
Homologue	*A*. *nancymaae*	51,993	27,298	953	4.77	199.67	6,981
	*C*. *jacchus*	51,124	27,482	962	4.82	199.55	6,937
	*C*. *syrichta*	49,121	26,909	949	4.90	193.62	6,648
	*O*. *princeps*	52,318	34,584	941	4.83	194.94	8,786
	*O*. *curzoniae*	288,876	34,488	312	2.07	150.75	31,928
RNAseq	TransDecoder	16,521	25,673	945	6.61	670.89	3,789
Integration	Maker	24,410	32,363	1,382	8.20	244.33	4,214
BUSCO	C: 98.4% [S: 93.7%, D: 4.7%], F: 0.4%, M: 1.2%, n: 255						

Note: C, Complete BUSCOs; S, Complete and single-copy BUSCOs; D, Complete and duplicated BUSCOs; F, Fragmented BUSCOs; M, Missing BUSCOs; n, number.

Functional annotation of the predicted genes in the *L*. *oiostolus* genome was performed by alignment to the UniProt, Pfam, GO, KEGG, KEGG pathway, Interproscan, and NR databases using Diamond BLASTP v2.11.0 (parameter -evalue 1e-5)^27^ and KOBAS v3.0^28^. Motifs, conserved sequences, and domains were annotated using InterProScan v5.33-72.0^29^ and hmmscan v3.1 (parameter e-value 0.01)^30^. A total of 22,295 protein-encoding genes were functionally annotated in the *L*. *oiostolus* genome (Table S7).

### Identification of non-coding RNA genes

Noncoding RNAs, including small nuclear RNAs (snRNA) and microRNAs (miRNA), were identified using INFERNAL v1.1.2^31^ based on the Pfam database^32^. Predictions of tRNAs were generated using tRNAscan-SE v1.23^33^. The rRNAs and their subunits were predicted using RNAmmer v1.2^34^. 463 miRNAs, 2,293 snRNAs, 1,053 tRNAs, and 541 rRNAs were predicted in the *L*. *oiostolus* genome (Table S8).

## Data Records

The assembled genome has been deposited at DDBJ/ENA/GenBank under the accession JAWMBE000000000^35^. The raw reads were also deposited at Sequence Read Archive (SRA) at NCBI, respectively^36^, under Bioproject PRJNA1026309. Data of the gene functional annotations, and repeat annotation had been deposited at figshare^37^.

## Technical Validation

### The quality control of genome

The BUSCO v4.1.4 (parameters: -evalue 1e-05)^38^ was used to assess the completeness and accuracy of the assembled genome. For the BUSCO analysis, 96.2% of genes were completely recalled, 93.3% were single copies, and 2.9% originated from duplication events (Table S9).

### Supplementary information

Supplementary Information

## Data Availability

All software used in this work is in the public domain, with parameters being clearly described in Methods. If no detail parameters were mentioned for a software, default parameters were used as suggested by developer.
