# Supplementary material for: Chromosome-scale genome assembly of Lepus oiostolus (Lepus, Leporidae)
Source: Sci Data. 2024 Feb 10;11:183. doi: 10.1038/s41597-024-03024-6 (PMC10858874; doi:10.1038/s41597-024-03024-6)
Supplement: Supplementary file 1 — Supplementary Information [file 41597_2024_3024_MOESM1_ESM.docx]

### Chromosome-scale genome assembly of *Lepus oiostolus* (*Lepus*, Leporidae)

Shuo Feng^1, 3, *^, Yaying Zhang^1, 3^, Zhaotong He^1^, Erning Xi^1^, Dafu Ru^2^, Jian Liang^1^ & Yongzhi Yang^2^

1. State Key Laboratory of Plateau Ecology and Agriculture, Qinghai University, Xining 810016, China

2. State Key Laboratory of Grassland Agro-Ecosystems, and College of Ecology, Lanzhou University, Lanzhou 730000, China

3. These authors contributed equally to this work: Shuo Feng, Yaying Zhang

*Corresponding author: Shuo Feng (fengshuo8894@126.com)

CONTENTS

[Supplementary Figure 1. K-mer (=19) depth analysis of the *L*. *oiostolus* genome. 3](#_Toc155186602)

[Supplementary Figure 2. Whole genome analysis of chromatin interactions at 500-kb resolution in *L*. *oiostolus* genome. 4](#_Toc155186603)

[Supplementary Figure 3. Comparison of genes of closely related species. 5](#_Toc155186604)

[Supplementary Table 1. Subreads statistics table. 6](#_Toc155186605)

[Supplementary Table 2. Illumina short-read sequencing data. 7](#_Toc155186606)

[Supplementary Table 3. Statistics of genome size estimation by 19-mer analysis. 8](#_Toc155186607)

[Supplementary Table 4. Assessment of genome consistency based on Illumina reads. 9](#_Toc155186608)

[Supplementary Table 5. Statistics of Hi-C reads of the *L*. *oiostolus*. 10](#_Toc155186609)

[Supplementary Table 6. Statistics of Hi-C mapping of the *L*. *oiostolus*. 11](#_Toc155186610)

[Supplementary Table 7. Summary statistics of noncoding RNA. 12](#_Toc155186611)

[Supplementary Table 8. Summary statistics of functional annotated protein-coding genes. 13](#_Toc155186612)

[Supplementary Table 9. BUSCO analysis of genome assembly (mammalia_odb10) 14](#_Toc155186613)


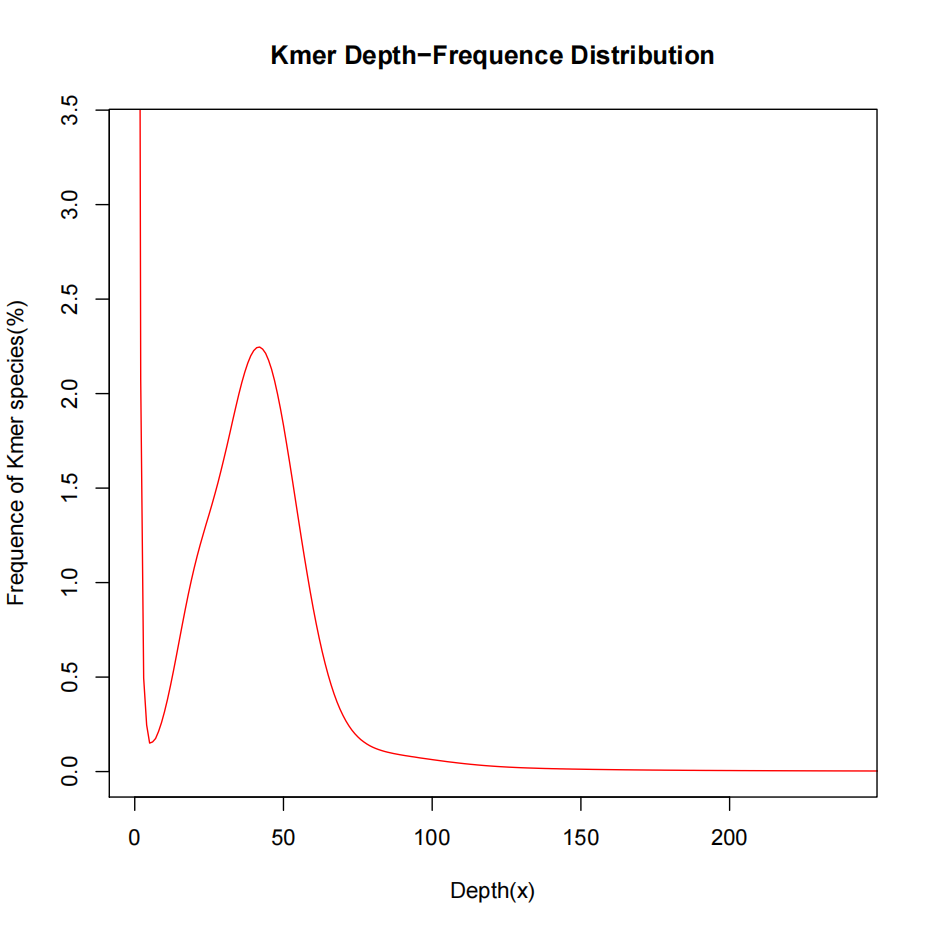


# Supplementary Figure 1. K-mer (=19) depth analysis of the *L*. *oiostolus* genome.


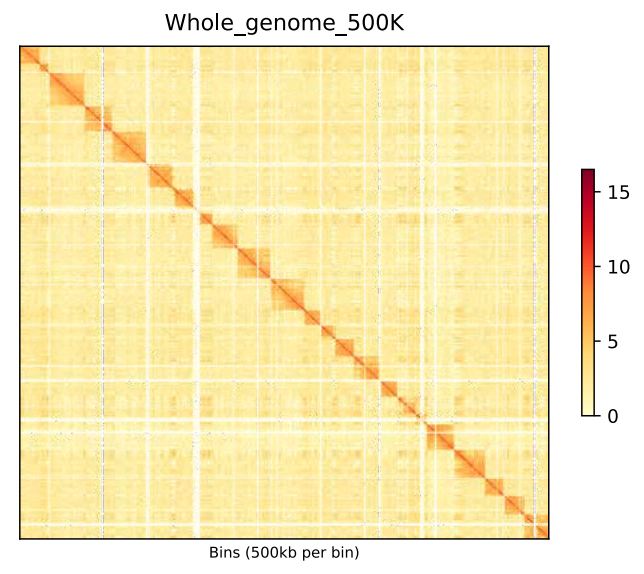


# Supplementary Figure 2. Whole genome analysis of chromatin interactions at 500-kb resolution in *L*. *oiostolus* genome.





# Supplementary Figure 3. Comparison of genes of closely related species.

# Supplementary Table 1. Subreads statistics table.

| **sample** | **number** | **subreads base (bp)** | **Average (bp)** | **N50 (bp)** | **GC (%)** |
| --- | --- | --- | --- | --- | --- |
| *L. oiostolus* | 3,913,830 | 79,026,003,674 | 20,191.48 | 20,168 | 0.43 |

# Supplementary Table 2. Illumina short-read sequencing data.

| **Sample name** | **Total reads** | **Total bases** | **GC (%)** | **Q20 (%)** | **Q30 (%)** |
| --- | --- | --- | --- | --- | --- |
| Raw data | 1,180,981,784 | 177,147,267,600 | 44.85% | 96.99% | 92.34% |
| Clean data | 1,180,981,764 | 177,147,264,600 | 44.85% | 96.99% | 92.34% |

# Supplementary Table 3. Statistics of genome size estimation by 19-mer analysis.

| **K-mer** | **Number** | **Depth** | **Genome Size (M)** | **Size (G)** | **X** | **Heterozygous Ratio (%)** | **Duplication Ratio (%)** |
| --- | --- | --- | --- | --- | --- | --- | --- |
| 19 | 119,285,326,993 | 41.95 | 2,833.96 | 177.15 | 62.51 | 0.72 | 36.55 |

# Supplementary Table 4. Assessment of genome consistency based on Illumina reads.

| **Type** | **Number** |
| --- | --- |
| Map rate | 99.99% |
| Average depth | 61.82 |
| Coverage | 99.99% |

# Supplementary Table 5. Statistics of Hi-C reads of the *L*. *oiostolus*.

| **Type** | **Total reads** | **Total bases** |
| --- | --- | --- |
| cleandata | 1,175,660,255 | 352,698,076,500 |

# Supplementary Table 6. Statistics of Hi-C mapping of the *L*. *oiostolus*.

| **Item** | **CXZ** |
| --- | --- |
| Raw Paired-end Pairs | 1,175,660,255 |
| Unique Mapped Paired-end Pairs | 748,168,828（63.6%） |
| Dangling End Paired-end Pairs | 105,151（0.008%） |
| Self-Circle Paired-end Pairs | 1,321,160（0.11%） |
| Valid Paired-end Pairs | 555,946,606（74.31%） |

# Supplementary Table 7. Summary statistics of functional annotated protein-coding genes.

| **Values** | **Total** | **Uniprot** | **Pfam** | **GO** | **KEGG** | **Pathway** | **Interproscan** | **NR** |
| --- | --- | --- | --- | --- | --- | --- | --- | --- |
| Number | 22,295 | 20,838 | 19,072 | 19,264 | 15,217 | 8,747 | 21,253 | 19,954 |
| Percentage (%) | 100 | 93.46 | 85.54 | 86.41 | 68.25 | 39.23 | 95.33 | 89.50 |

# Supplementary Table 8. Summary statistics of noncoding RNA.

| Type |  | Copy | Average length(bp) | Total length(bp) | % of genome |
| --- | --- | --- | --- | --- | --- |
| miRNA |  | 463 | 85 | 39,440 | 0.001381 |
| tRNA |  | 1,053 | 77 | 81,505 | 0.002854 |
| rRNA | rRNA | 541 | 372 | 201,123 | 0.007044 |
|  | 18S | 39 | 1,645 | 64,145 | 0.002246 |
|  | 28S | 65 | 1,316 | 85,552 | 0.002996 |
|  | 5.8S | 36 | 152 | 5,489 | 0.000192 |
|  | 5S | 401 | 115 | 45,937 | 0.001609 |
| snRNA | snRNA | 2,293 | 121 | 277,793 | 0.009729 |
|  | CD-box | 417 | 89 | 37,239 | 0.001304 |
|  | HACA-box | 431 | 133 | 57,338 | 0.002008 |
|  | splicing | 1,410 | 126 | 177,300 | 0.006209 |
|  | scaRNA | 35 | 169 | 5,916 | 0.000207 |

# Supplementary Table 9. BUSCO analysis of genome assembly (mammalia_odb10).

| **Item** | **Number** | **Percent (%)** |
| --- | --- | --- |
| Complete BUSCOs (C) | 8,873 | 96.2 |
| Complete and single-copy BUSCOs (S) | 8,608 | 93.3 |
| Complete and duplicated BUSCOs (D) | 265 | 2.9 |
| Fragmented BUSCOs (F) | 79 | 0.9 |
| Missing BUSCOs (M) | 274 | 2.9 |
| Total BUSCO groups searched | 9,226 | 100.0 |
